# ISOLATED TONSIL TUBERCULOSIS

**DOI:** 10.4103/0970-2113.45284

**Published:** 2008

**Authors:** Surya Kant, Sanjay Kumar Verma, Sanjay

**Affiliations:** 1 Professor, King George’s Medical University, Lucknow (India); 2 Senior Resident, King George’s Medical University, Lucknow (India); 3 Junior Resident, King George’s Medical University, Lucknow (India)

**Keywords:** Tuberculosis, Tonsil

## Abstract

The occurrence of tuberculosis of the upper respiratory tract including oral cavity has become uncommon. Isolated tuberculosis of tonsil in the absence of active pulmonary tuberculosis is very rare clinical entity. Here is a report of tonsil tuberculosis, presented with complaints of sore throat.

## INTRODUCTION

Approximately two per cent of patients with active pulmonary tuberculosis show evidence of upper respiratory tract involvement^1^. Although the most common site is larynx and other structures such as tongue, palate, tonsils, pharynx and buccal mucosa may also be involved. Primary tuberculosis of the tonsil in the absence of active pulmonary tuberculosis is rare^2^ which has prompted us to report this case.

## CASE REPORT

A 55 Years old male presented with history of sore throat and difficulty in swallowing of solid food for two months. There was no history of cough, fever, hoarseness of voice, vomiting and regurgitation of food. Examination of oropharyngeal cavity showed an ulcer over anterior pillor of left tonsil about 8 × 8 mm in dimension. Rest of the oral cavity was apparently normal on gross appearance. There was no lymphadenopathy, dental caries or damaged teeth. The examination of chest was normal. The patient was HIV seronegative. Ultrasonographic and barium meal studies did not reveal any abnormality. PPD was positive with indurations of 18 × 20 mm. Punch biopsy was taken from the ulcerative growth on left tonsil. Histopathological examination of the biopsy revealed presence of tubercular granuloma characterized by epitheloid cells, langhans type of giant cells and mononuclear inflammatory cells (Figure I). The acid-fast bacilli were not be detected and culture was positive for Mycobacterium Tuberculosis. Antituberculosis treatment with isoniazid 300 mg, rifampicin 450 mg and pyrazinamide 1500 mg was started for two months followed by rifampicin and isoniazid for four months. He had responded well to the treatment. During follow up, sore throat and difficulty in swallowing was reduced. On gross appearance, the ulcers on anterior pillor of left tonsil get resolved.

**Fig 1:**
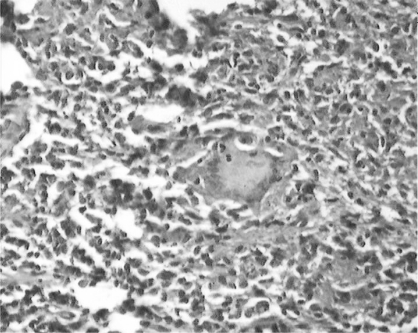
Biopsy examination revealed presence of tubercular granuloma characterized by epitheloid cells, langhans type of giant cells and mononuclear inflammatory cells.

## DISCUSSION

Extra pulmonary tuberculosis (TB) represents approximately 25 % of overall tubercular morbidity^3^. Among extra pulmonary tuberculosis (EPTB), most common is lymph node tuberculosis while other forms are: pleural tuberculosis, skeletal tuberculosis, CNS tuberculosis, abdominal tuberculosis, genito-urinary tuberculosis, miliary tuberculosis, tubercular pericarditis are also seen. Tuberculosis of the oral cavity is uncommon and tonsillar forms are extremely rare^4^.

Tonsillar TB commonly presents with sore throat and cervical lymphadenopathy^5^. This presentations as well as abnormal tonsillar finding, make it difficult to differentiate tonsillar tuberculosis from a malignant tumor.

Diagnosis of tonsillar tuberculosis is based on histopathological findings and the identification of tubercle bacilli^6^–^8^. Treatment is in the form of anti-tuberculosis therapy. Differential diagnosis of oral and pharyngeal tuberculosis includes traumatic ulcers, aphthous ulcers, hematological disorders, actinomycosis, syphilis, midline granuloma, Wegner′s disease and malignancy^6^–^7^,^9^.

In our case, ulceration over left tonsil and difficulty in swallowing of solid foods evoked the suspicion of malignancy. The clinician should remain alert to the possibility of tuberculosis especially in older patients and in developing countries like India where the incidence of tuberculosis is quite high.
